# All-dry flip-over stacking of van der Waals junctions of 2D materials using polyvinyl chloride

**DOI:** 10.1038/s41598-022-26193-z

**Published:** 2022-12-19

**Authors:** Momoko Onodera, Yusai Wakafuji, Taketo Hashimoto, Satoru Masubuchi, Rai Moriya, Yijin Zhang, Kenji Watanabe, Takashi Taniguchi, Tomoki Machida

**Affiliations:** 1 Institute of Industrial Science, University of Tokyo, 4-6-1 Komaba, Meguro, Tokyo, 153-8505 Japan; 2 Riken Technos Corporation, Waterras Tower, 2-101 Kanda-Awajicho, Chiyoda, Tokyo, 101-8336 Japan; 3 Research Center for Functional Materials, National Institute for Materials Science, 1-1 Namiki, Tsukuba, 305-0044 Japan; 4 International Center for Materials Nanoarchitectonics, National Institute for Materials Science, 1-1 Namiki, Tsukuba, 305-0044 Japan

**Keywords:** Graphene, Two-dimensional materials

## Abstract

We demonstrated an all-dry polymer-to-polymer transfer technique for two-dimensional (2D) crystal flakes using a polyvinyl chloride (PVC) layer deposited on a piece of polydimethylsiloxane (PDMS). Unexpectedly, the pickup/release temperatures were modified in wider temperature range simply by changing the thickness of the PVC layer than changing the plasticizer ratio. Utilizing the difference in the pickup/release temperatures depending on the PVC film thickness, 2D flakes were transferred from a thicker PVC film to a thinner one. This polymer-to-polymer transfer technique can be utilized to flip over van der Waals heterostructures. As a demonstration, we fabricated a mountain-like stacked structure of hexagonal boron nitride flakes using the flip-over stacking technique. Finally, we compared the results of thermomechanical analysis with the pickup/release temperatures of the PVC/PDMS stamp. The PVC was revealed to be at the glass transition and in the viscoelastic flow regimes when the 2D flakes were picked up and dry released, respectively. Our polymer-to-polymer transfer method facilitates flip-over van der Waals stacking in an all-dry manner, expanding the possibility of 2D materials device fabrications.

## Introduction

Two-dimensional (2D) materials science has developed in tandem with the advancement of transfer techniques utilizing polymers. Many techniques for transferring 2D materials have been devised^1^, and these allow 2D flakes to be deposited onto specific substrates or vertically stacked into van der Waals (vdW) heterostructures^2^. In general, vdW heterostructures are fabricated either by means of bottom-up or top-down methods. In the former, 2D flakes are deposited on a substrate one by one, from the bottom layer to the top layer^3–5^. In the latter, the topmost 2D flake is first picked up by an adhesive polymer stamp, and then, the 2D flakes are successively picked up by vdW force between the 2D flakes, and thus the stack is fabricated from the top layer to the bottom layer^6–8^. The advantage of top-down transfer is that it can be used to prepare high-quality vdW devices because the lower 2D layers are protected from direct contact with the polymer. Furthermore, top-down pickup is necessary for the tear-and-stack technique^9^, which is essential for the fabrication of state-of-the-art twist-angle-controlled multilayer devices^10^. On the other hand, there are some restrictions on the device structures that can be created using the top-down approach because flakes are picked up from larger to smaller flakes, and a thin 2D flake tends to get wrinkled when it is picked up first. Scanning tunneling microscopy and angle-resolved photoemission spectroscopy^11,12^ often require device structures that cannot be fabricated using top-down transfer alone, such as a structure having a conductive monolayer on top. To overcome this limitation, previous studies utilized techniques involving flipping over the vdW stack using polydimethylsiloxane (PDMS)^11^, polypropylene carbonate (PPC)^13,14^, and Elvacite^15^. However, the PDMS technique requires dexterous operation upon picking up flakes, and the PPC and Elvacite techniques require high-temperature annealing or immersion into organic solvents to remove the stamp polymer. The most desirable is an easy-to-operate top-down transfer technique that enables pickup, flip-over, and release of 2D flakes in an all-dry manner.

Polyvinyl chloride (PVC) has recently been recognized as a versatile polymer for the pickup and dry-release of 2D crystal flakes^16,17^. The advantages of PVC are (1) strong adhesion to both the surface and edge of 2D flakes, (2) the adhesion between PVC and 2D flakes can be changed significantly with temperature, and (3) 2D flakes can be released onto a SiO_2_/Si substrate without being melted, i.e., the transfer is conducted in an all-dry manner. These characteristics are related to the viscoelasticity of PVC. In general, the viscoelasticity of a polymer depends on the degree of polymerization, amount of plasticizers added, film thickness, applied pressures, and so on. Transfer of 2D flakes is expected to be realized between two polymer stamps having different adhesion owing to the difference in viscoelasticity.

In this study, we demonstrated the all-dry flip-over stacking of 2D materials using a PVC/PDMS stamp. 2D flakes are first picked up by a PVC/PDMS stamp and then transferred to another stamp, which we call the polymer-to-polymer transfer of 2D materials. The key point of the polymer-to-polymer transfer is the difference in the pickup/release temperatures of the PVC stamp. We found that pickup/release temperatures are modified by PVC film thickness, rather than by plasticizer content. Thicker PVC films exhibit lower pickup and release temperatures, hence 2D flakes can be transferred from a thicker film to a thinner one utilizing the difference in adhesion. The polymer-to-polymer transfer enables us to flip the 2D stack upside down in an all-dry manner, thereby widening the potential applications of top-down transfer.

**Figure 1:**
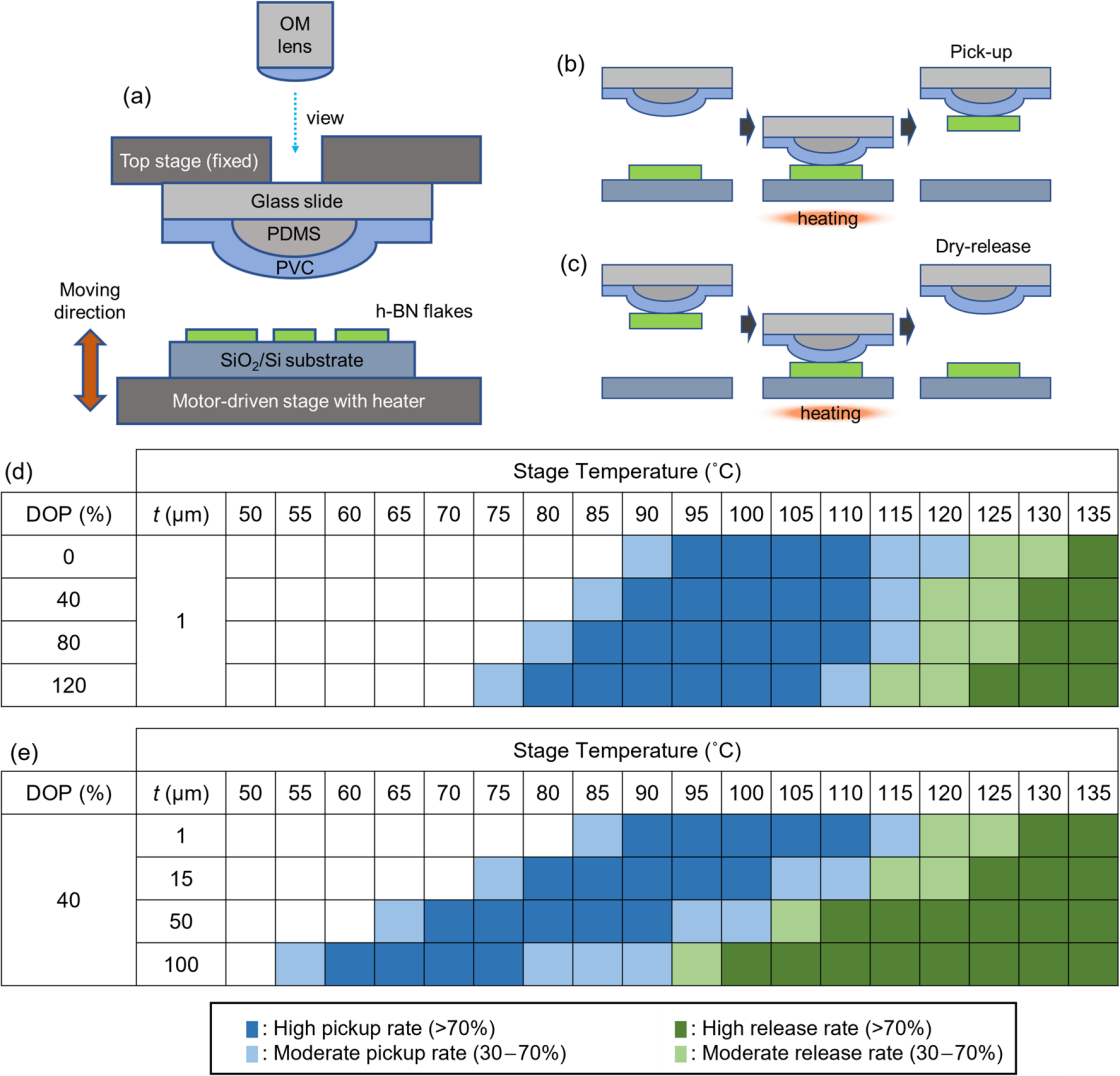
(**a**) Schematic of our motor-driven sample stage setup for the pickup of *h-*BN flakes. During operation, a SiO_2_/Si substrate with exfoliated *h-*BN flakes is fixed on the motor-driven stage (lower stage), and a PVC/PDMS stamp is fixed to the upper stage using a vacuum chuck. The PVC/PDMS stamp is brought into contact with or detached from the SiO_2_/Si substrate by moving the lower stage up or down, respectively. The temperature of the lower stage is controlled by a heater. (**b,c**) Schematics of (**b**) pickup and (**c**) dry-release of an *h-*BN flake from a SiO_2_/Si substrate. (**d,e**) Test results for pickup/release using PVC/PDMS stamps. (**d**) DOP content dependence of pickup/release for 1-μm PVC films. (**e**) Film-thickness dependence of pickup/release for PVC with 40% DOP. Blue (green) cells indicate conditions under which pickup (release) is possible.

First, we conducted experiments to evaluate the effect of the plasticizer content of PVC on the pickup temperature of PVC/PDMS stamps. The addition of plasticizers is the most frequently used way to modulate the glass transition temperature *T*_g_ of polymers, with *T*_g_ being lowered by the presence of plasticizer molecules. Previous literature reported a decrease in *T*_g_ by 20–30 °C in response to the addition of plasticizer (40 wt.%) to the PVC^18–20^. As the *T*_g_ of PVC depends on various factors, the effect of plasticizers should be experimentally investigated under practical 2D flake pickup conditions, in which a thin PVC film is laid on a PDMS dome. We conducted testing of the pickup and release of 2D flakes on SiO_2_/Si substrates using PVC/PDMS stamps with varying plasticizer contents. A plasticizer (dioctyl phthalate, DOP) was mixed with PVC powder, and the mixture was dissolved in cyclohexanone. To prepare a PVC stamp for 2D flake transfer, a 1-μm-thick PVC layer was fabricated from the PVC/DOP/cyclohexanone solution (Figs. S1 and S2). The PVC layer was then laid on a dome-shaped piece of PDMS on a glass slide (Fig. S3). In this study, we performed tests using hexagonal boron nitride (*h-*BN) grown by the high-pressure and high-temperature method^21^ because it is the most widely used 2D material in the 2D materials research field as an ideal substrate and capping layer for various 2D materials^4,22^. The *h-*BN crystal was mechanically exfoliated onto a SiO_2_/Si substrate. The SiO_2_/Si substrate with exfoliated *h-*BN flakes was placed on a lower stage, and the PVC/PDMS stamp on a glass slide was placed on an upper stage (Fig. 1a). To perform pickup and release of the 2D flakes using the stamp, the lower stage was motor driven. The PVC/PDMS stamp was brought into contact with the *h-*BN flakes on the SiO_2_/Si substrate and then the stamp was detached from the substrate. We repeated this process with changing the stage temperature *T* and recorded whether the flake was picked up (Fig. 1b) or released (Fig. 1c) onto the substrate.

Figure 1d summarizes the results of the pickup tests. Blue (green) cells indicate temperature and DOP-content conditions under which the flakes were picked up (released). The pickup and release temperatures, *T*_pickup_ and *T*_release_, of PVC decrease as the plasticizer content increases. The magnitude of this shift in *T*_pickup_ is, however, about a quarter of that in *T*_g_ reported in the literature^18–20^. This discrepancy is attributed to the difference in the thickness of the PVC samples. In other words, the data in the literature was obtained using thick (1 mm or greater) PVC samples whereas our PVC layers were ~ 1 μm thick. It is known that the *T*_g_ of a polymer film depends on the film thickness^23^, while the information on *T*_g_ of the micrometer-thick PVC is still lacking. Therefore, we conducted pickup tests with varying PVC film thicknesses, keeping the DOP content constant (40%). Figure 1e shows the results of the pickup tests conducted using PVC/PDMS stamps having different PVC film thicknesses. *T*_pickup_ and *T*_release_ were greatly reduced for the thicker PVC films, approaching the *T*_g_ value expected from the data in the literature. For the 100-μm-thick 40% DOP film sample, *T*_pickup_= 60–75 °C. It is notable that, although the composition of the PVC films is the same, *T*_pickup_ and *T*_release_ was different for films of different thicknesses. Tuning of *T*_pickup_ and *T*_release_ by film thickness is more convenient than by plasticizer content because we do not have to prepare multiple PVC/plasticizer solutions.

**Figure 2:**
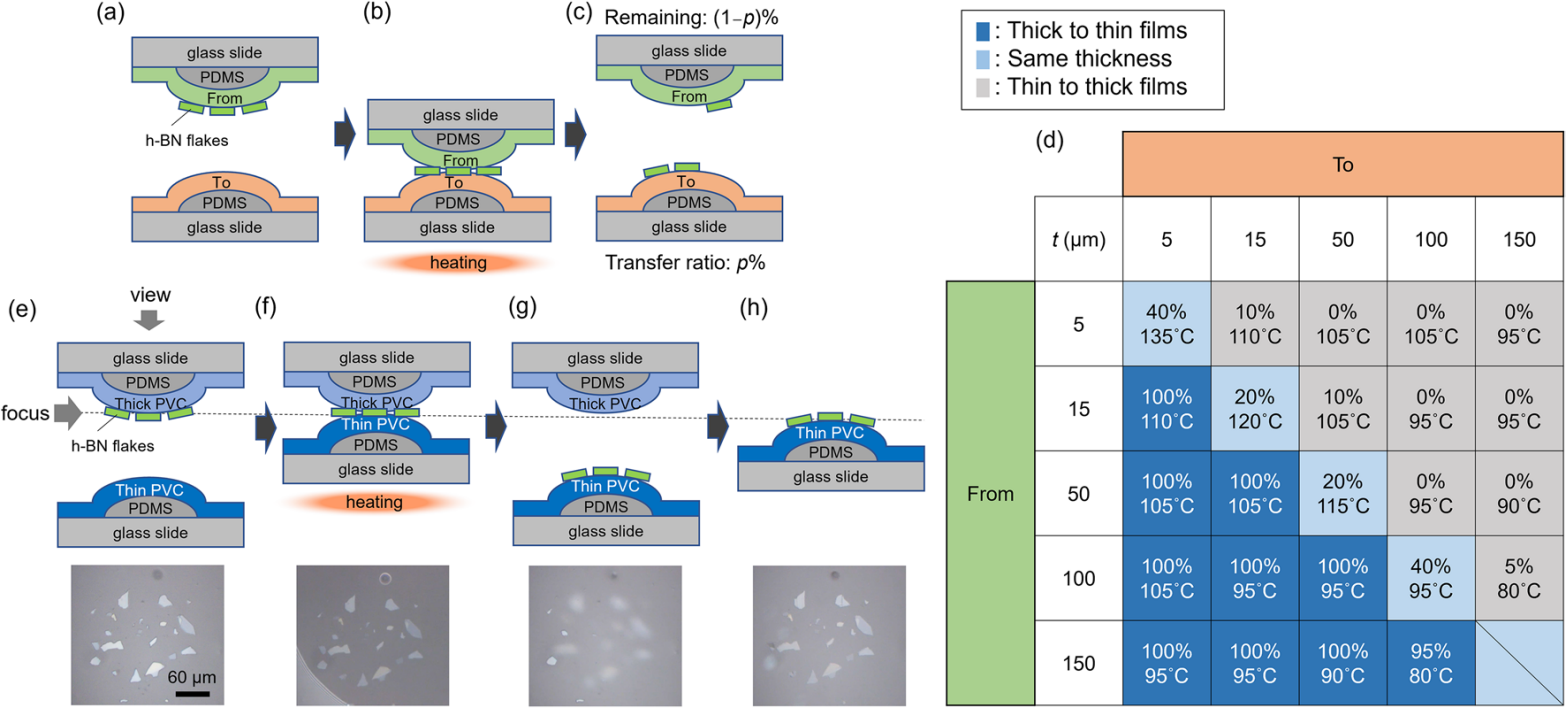
(**a–c**) Schematics of the polymer-to-polymer transfer testing. (**a**) The “from”-stamp with *h-*BN flakes was set above the “to”-stamp. (**b**) The from- and to-stamps were brought into contact and then (**c**) detached at a certain temperature, and the *h-*BN flake transfer ratio and stage temperature were recorded. (**d**) Polymer-to-polymer transfer test results (see text). (**e–h**) Schematics and optical micrographs of polymer-to-polymer transfer of *h-*BN flakes. (**e**) A thin PVC/PDMS stamp with adhered *h-*BN flakes was positioned above a thick PVC\PDMS stamp, and the two stamps were (**f**) brought into contact and then (**g**) detached at a certain temperature. (**h**) *h-*BN flakes after transfer to the thick PVC/PDMS stamp. The dotted line indicates the focal plane of the microscope during micrograph acquisition.

In this study, we focused on the large film-thickness-dependent shift in *T*_pickup_ and *T*_release_. Because *T*_pickup_ and *T*_release_ are strongly dependent on thickness, *T*_release_ for a thick PVC film can be as low as *T*_pickup_ for a thin PVC film. For example, *T* = 100 °C is in the range of *T*_pickup_ for 15-μm-thick PVC and is in the range of *T*_release_ for 100-μm PVC (Fig. 1e). Thus, it is expected that 2D flakes will be transferred from 15-μm PVC to 100-μm PVC at *T* = 100 °C. To confirm this hypothesis, we conducted transfer experiments, moving *h-*BN flakes between different PVC stamps (polymer-to-polymer transfer) while varying the PVC film thickness (Fig. 2). First, *h-*BN flakes on a SiO_2_/Si substrate were sequentially picked up by a PVC stamp attached to the upper stage (“from”-stamp), with each flake occupying a separate area on the stamp surface. Then, another PVC stamp without any *h-*BN flakes was placed on the lower stage (“to”-stamp) (Fig. 2a). The stage temperature was first set to a value below *T*_pickup_ (40–50 °C), and contact was made between the “from”- and “to”-stamps with the stage speed *v* set to 500 μm/s, and then the stamps were slowly detached at *v* = 20 μm/s (Fig. 2b,c). We repeated this process, increasing the stage temperature until flakes were no more transferred to the to-stamp. The temperature corresponding to the maximum flake transfer was recorded, along with the flake transfer ratio (%), i.e., the number of flakes transferred to the to-stamp relative to the initial number on the from-stamp (Fig. 2c). The results are summarized in Fig. 2d. The left and top sides of the chart indicate the thicknesses of the PVC in the from- and to-stamps, respectively. The percentage and temperature in each cell indicate the transfer ratio and temperature at which the transfer ratio was saturated.

First, we conducted transfer tests from thicker to thinner films (Fig. 2d, dark blue cells). Figure 2e–h show an example of this transfer process. As expected from the results of the pickup tests (Fig. 1e), the transfer ratio was almost 100% within all the range of thicknesses investigated. The transfer temperature *T*_transfer_ was 80–110 °C, which approximately corresponds to *T*_pickup_ for the thinner film and *T*_release_ for the thicker film. This result demonstrates that adhesion between 2D flakes and PVC is modulated by the film thickness, and 2D flakes are transferred from less adhesive to more adhesive PVC stamps with a high transfer rate. We also tested the polymer-to-polymer transfer between PVC films of the same thickness (light blue cells) and from thinner to thicker films (gray cells), for which polymer-to-polymer transfer is unlikely to occur. As expected, the transfer rate in these cases was not high. The transfer ratio between films of the same thickness was less than 40%, and the transfer ratio was less than 10% from thinner to thicker films. This result confirms that polymer-to-polymer transfer only occurs from thicker to thinner PVC owing to the difference between the PVC–2D flake adhesion forces of the films. Several technical aspects related to the conditions required for polymer-to-polymer transfer are discussed in the Supplementary data.

**Figure 3:**
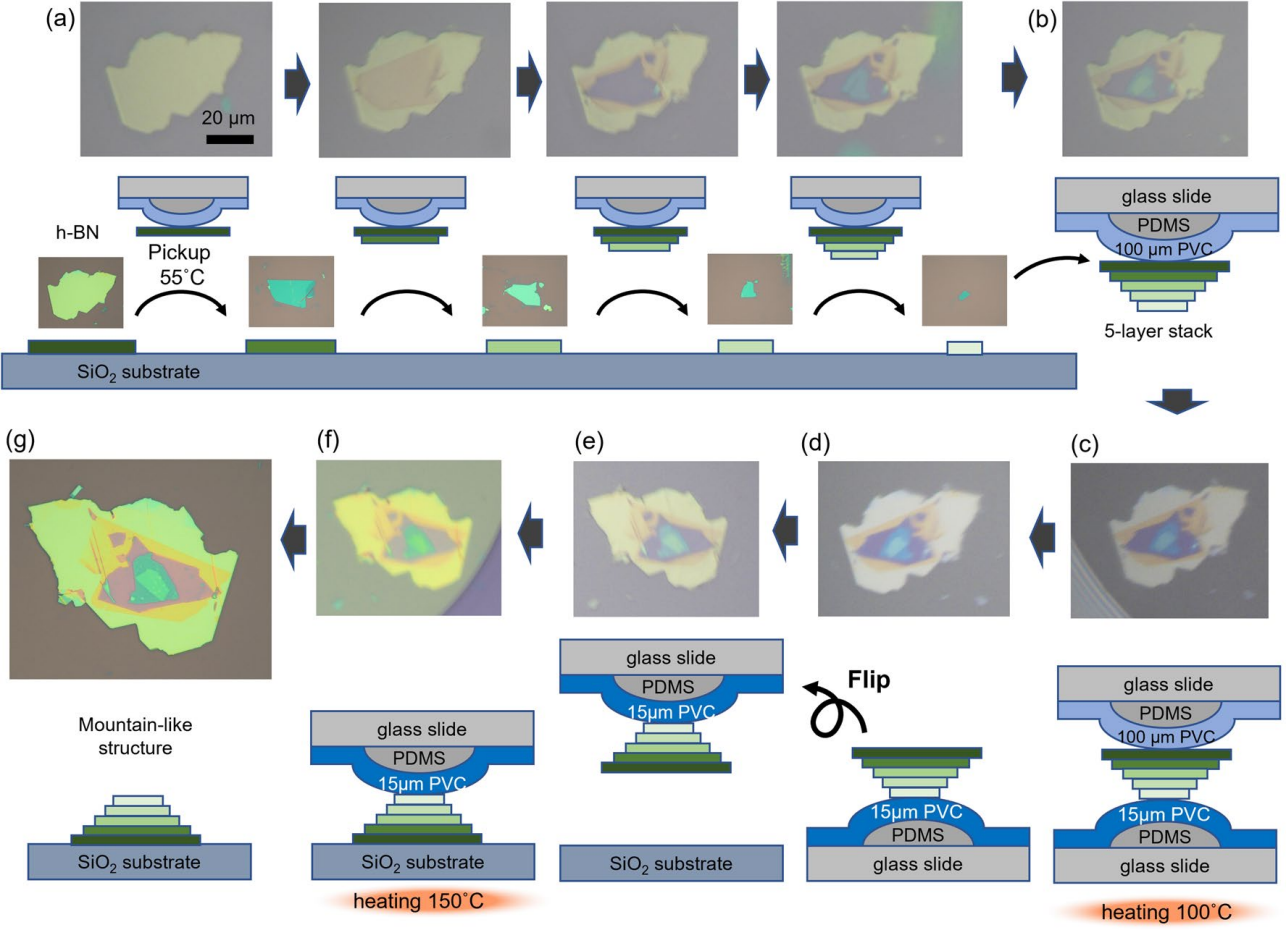
Demonstration of flip-over stacking. (**a**) Five *h-*BN flakes on a SiO_2_/Si substrate were picked up by a 100-μm-PVC/PDMS stamp at *T* = 55 °C, starting with the largest and proceeding to sequentially smaller flakes, and hence (**b**) an inverse-mountain-like stacked structure was created. (c) The stack was brought into contact with and (**d**) transferred to a 15-μm-PVC/PDMS stamp at *T* = 100 °C. (**e**) The 15-μm-PVC/PDMS stamp was inverted and (**f**) brought into contact with a SiO_2_/Si substrate before being slowly detached at *T* = 150 °C. (**g**) The stack was dry-released on the substrate, and a mountain-like layered *h-*BN structure was obtained. The images in (**a–f**) are top-view micrographs acquired through the upper glass slide.

Polymer-to-polymer transfer is useful for procedures involving flipping vdW stacks upside-down. As a demonstration, we fabricated a mountain-like *h-*BN stack structure via polymer-to-polymer transfer (Fig. 3). For this demonstration, 15- and 100-μm PVC films were selected, considering their *T*_pickup_, *T*_release_, film toughness, and ease of handling. First, five *h-*BN flakes on a SiO_2_/Si substrate were sequentially picked up; The first flake was picked up owing to adhesion between the 100-μm-PVC/PDMS stamp and the flake, and the vdW forces between *h-*BN layers mediated the pickup of subsequent flakes (Fig. 3a). At this point, the flake size decreased from the top to the bottom, and the *h-*BN vdW structure took an inverted mountain-like form (Fig. 3b). Then, the *h*-BN stack on the 100-μm-PVC/PDMS stamp was brought into contact with the 15-μm-PVC/PDMS stamp on the lower stage at *T* = 100 °C (Fig. 3c). The stack on the 15-μm-PVC/PDMS stamp (Fig. 3d) was then inverted and fixed to the upper stage (Fig. 3e). Finally, the *h-*BN stack was dry-released onto a SiO_2_/Si substrate at *T* = 150 °C (Fig. 3f), and the mountain-like *h-*BN stack structure, in which the flake size increased from the top to the bottom, was obtained (Fig. 3g). The surface of the stacked structure can be cleaned by high temperature annealing (Fig. S4). This flip-over stacking is also applicable for other 2D materials such as graphite and MoS_2_ (Fig. S5).

**Figure 4:**
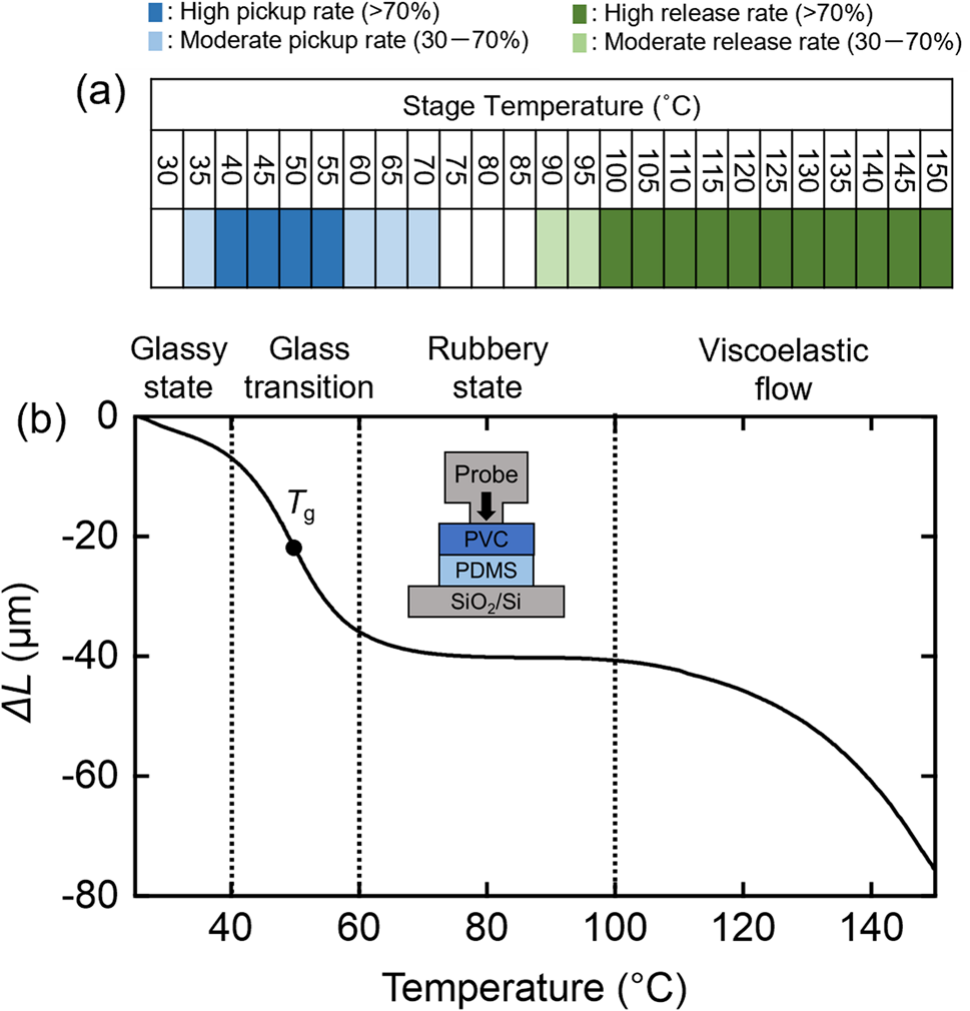
(**a**) Results of the pickup tests on the 130-μm-PVC/PDMS stamp at different temperatures. The PVC layer was fabricated by compressing multiple layers of 10-μm-PVC-based food wrap (~ 28% plasticizer). (**b**) TMA results for 130-μm-PVC/PDMS stamp. The vertical axis shows the probe displacement Δ*L.* Inset: schematic of the TMA apparatus.

The advantage of our technique is that all the processes of pickup, flip-over, and release are conducted in an all-dry manner. To give an insight on the pickup/release mechanism, we discuss *T*_pickup_ and *T*_release_ from the viewpoint of the static softening behavior of PVC/PDMS, based on thermomechanical analysis (TMA) (Fig. 4). TMA involves measurement of the displacement of the polymer under a constant pressure. The schematic of the TMA apparatus is shown in the inset of Fig. 4b. A probe made of SiO_2_ (diameter ~ 0.5 mm) was pressed against the sample such that the probe pressure was maintained at 0.9 mN. The displacement of the probe Δ*L* was recorded as the sample temperature was varied from 25 to 150 °C. Figure 4b shows the TMA results for a 130-μm PVC film laid on a 430-μm PDMS sheet; Δ*L* decreases with *T*, and there are two obvious reduction regions in Δ*L* at *T* = 40–60 °C and *T* > 100 °C. As the temperature increases, the polymer is transformed from the glassy state to a glass transition, rubbery state, a viscoelastic flow region, and toward the liquid flow region of its phase diagram^24^, as indicated in Fig. 4b (note that the liquid flow region was not observed in the temperature range used in this study). To compare the TMA data with *T*_pickup_ and *T*_release_, we show the results of the pickup tests on the 130-μm PVC layer deposited on a PDMS dome in Fig. 4a. It was found that the pickup region (blue cells) corresponds to the glass transition region, where the polymer transitions from a solid-like state to a rubber-like state. It is known that the viscosity/elasticity ratio is maximized in the vicinity of *T*_g_^25^, and this is thought to correlate with maximized mechanical adhesion for the polymer. Meanwhile, the release region (green cells) corresponds to the viscoelastic flow region of PVC/PDMS. In this region, the polymer approaches the fluid-like state and is unable to remain adhered to the 2D flakes. It is important to note that the 130-μm PVC film retains sufficient elasticity at temperatures up to 150 °C to allow the restoration of its original shape, and this is the principal reason why all-dry release can be achieved using PVC.

In conclusion, we demonstrated polymer-to-polymer transfer of 2D flakes using a PVC/PDMS stamp. *T*_pickup_ and *T*_release_ were modulated simply by controlling the film thickness, and thicker films were shown to possess lower *T*_pickup_ and *T*_release_ values than thinner films. The transfer of 2D flakes from thicker to thinner PVC layers was demonstrated, thanks to the difference between their abilities to adhere to 2D flakes. Our polymer-to-polymer transfer technique increases the possibilities for top-down transfer, and it represents a significant advancement in device fabrication techniques for 2D materials.

## Supplementary Information

Supplementary Information.

## Data Availability

The data that support the findings of this study are available from the corresponding author upon reasonable request.
