# Supplementary material for: All-dry flip-over stacking of van der Waals junctions of 2D materials using polyvinyl chloride
Source: Sci Rep. 2022 Dec 19;12:21963. doi: 10.1038/s41598-022-26193-z (PMC9763492; doi:10.1038/s41598-022-26193-z)
Supplement: Supplementary file 1 — Supplementary Information. [file 41598_2022_26193_MOESM1_ESM.pdf]

Supplementary data

All-dry flip-over stacking of van der Waals junctions of 2D materials using polyvinyl chloride

Momoko Onodera,[1*] Yusai Wakafuji,[1] Taketo Hashimoto,[2] Satoru Masubuchi,[1] Rai Moriya,[1] Yijin Zhang,[1] Kenji Watanabe,[3] Takashi Taniguchi,[1,4] Tomoki Machida[1*]

[1]Institute of Industrial Science, University of Tokyo, 4-6-1 Komaba, Meguro, Tokyo 153-8505, Japan

[2]Riken Technos Corporation, Waterras Tower, 2-101 Kanda-Awajicho, Chiyoda, Tokyo 101-8336, Japan

[3]Research Center for Functional Materials, National Institute for Materials Science, 1-1 Namiki, Tsukuba 305-0044, Japan

[4]International Center for Materials Nanoarchitectonics, National Institute for Materials Science, 1-1 Namiki, Tsukuba 305-0044, Japan

[*]E-mail: monodera@iis.u-tokyo.ac.jp and tmachida@iis.u-tokyo.ac.jp

PVC/PDMS stamp preparation

Polyvinyl chloride (PVC) powder (degree of polymerization, 680) was provided by Shin-Etsu Chemical Co., Ltd. Plasticizer (Dioctyl Phthalate, DOP) was dropped into PVC powder (~0.5-1.0 g) placed in a glass bottle (Mity Vial 28 mL, No.6, Maruemu Corporation), and the PVC and DOP were then mixed using a plastic rod. The mixture was maintained at 25 °C overnight, and then cyclohexane (2-5 mL) was poured into the bottle. The bottle was heated to 100 °C on a hotplate and occasionally shaken to promote dissolution. The PVC was dissolved within 24 h. Before use, the PVC solution was filtered by a syringe filter to remove particles. PVC films were then fabricated by one of the following two methods. The maximum thickness of the PVC films fabricated using method (1) was limited by the viscosity of the PVC solution (~15 μm). PVC films with thicknesses of up to ~150 μm were fabricated using method (2).

(1) Fabrication of PVC films using glass slides [Fig. S1]: Tape was used to cover two of the edges of a glass slide to act as a spacer, and PVC solution was dropped onto the center of the uncovered area of the slide using a disposable polyethylene pipet (DISPET 0.5 mL, Maruemu Corporation). The solution was spread over the glass using another glass slide. The PVC film was heated on a hotplate to $T = 80$ °C for more than 15 min for solidification. To increase the film thickness, the thickness of the spacer was adjusted by adding more layers of tape.

(2) Fabrication of PVC films using a tape frame structure [Fig. S2]: Multiple layers of tape were attached to a glass slide, and the center of the tape was cut out to create a tape frame structure. PVC solution was poured inside the frame and solidified on a hotplate.

A polydimethylsiloxane (PDMS) dome was prepared using a PDMS elastomer kit (SYLGARD®184, Dow Corning). A polymeric base and curing agent (10:1 by weight) were mixed and placed in a vacuum desiccator for 10 min to eliminate air bubbles. The liquid mixture was dropped on a PDMS sheet (PF-X4, 17 mm, Gel-Pak) that had been placed on a glass slide using a toothpick. The diameter of the dome was ~1-2 mm. The PDMS dome was solidified on a hotplate at $T = 130$ °C for 5 min.

To combine the PDMS dome and PVC film, a piece of tape was attached to each of the four edges of the PVC film, creating a PVC film window of ~4 × 4 mm^2. The PVC film was then

peeled off from the glass slide and transferred to a PDMS dome on a glass slide [Fig. S3]. Excess tape was trimmed using a rotary cutter. The PVC/PDMS stamp was baked in a vacuum oven at T = 80 °C overnight before use.

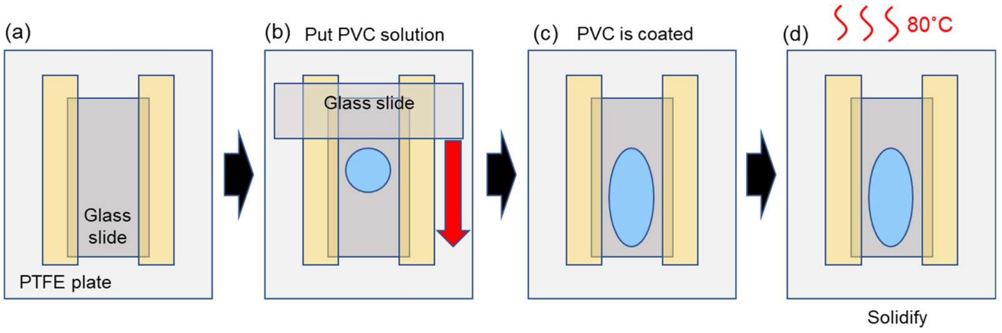

Fig. S1. Fabrication of PVC films using glass slides. (a) A glass slide is placed on a polytetrafluoroethylene (PTFE) sheet, and tape is attached to two of its sides. The tape functions as a spacer, determining the thickness of the film. (b) PVC solution is dropped onto the glass slide and (c) coated over the glass surface using another glass slide as a scraper. (d) The film is solidified on a hotplate at T = 80 °C.

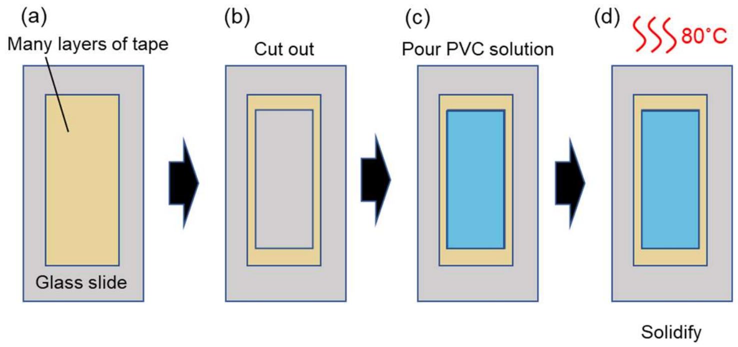

Fig. S2. Fabrication of PVC films using a tape frame structure. (a) Multiple layers of tape (stacking to a thickness of ~1 mm) are attached to a glass slide. (b) The center of the tape is extracted by cutting to create a frame structure. (c) PVC solution is poured into the tape pool and (d) solidified on a hotplate at T = 80 °C.

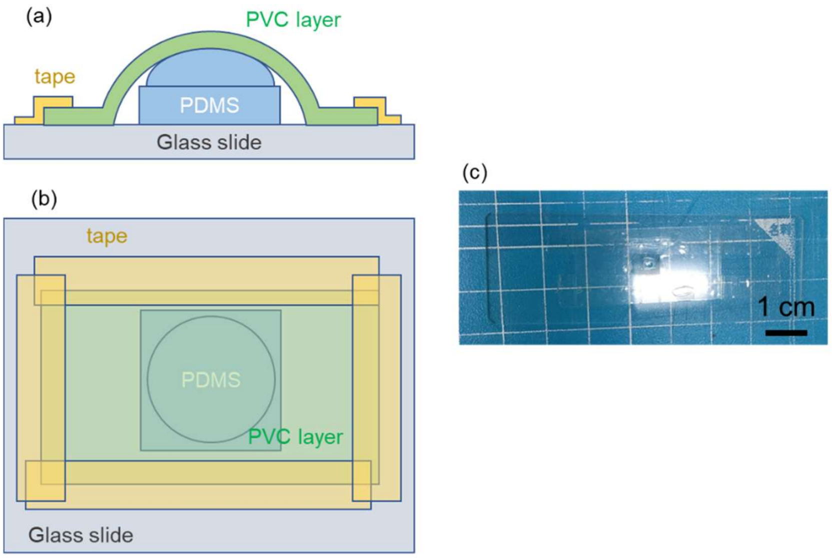

Fig. S3. (a) Side-view and (b) top-view schematics of the PVC/PDMS stamp. (c) Photograph of the PVC/PDMS stamp.

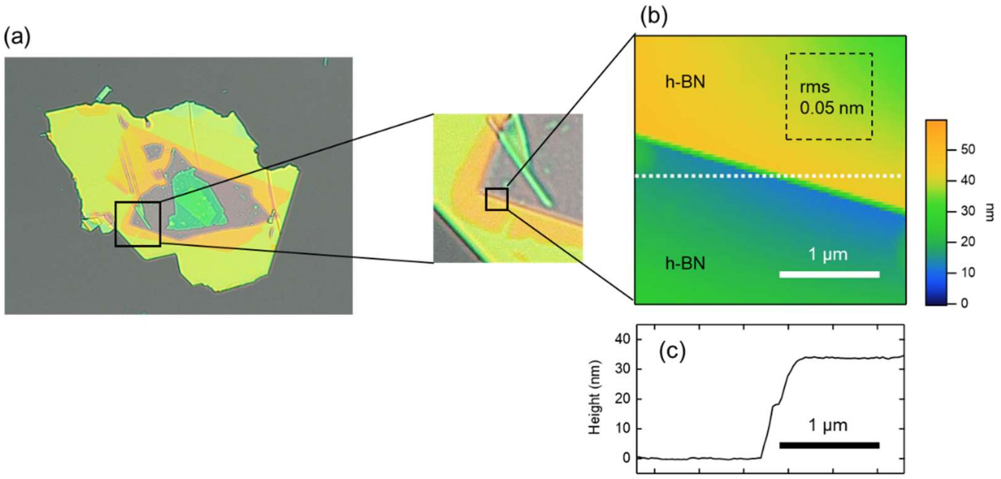

Fig. S4. (a) Photograph of the h-BN stack fabricated by the flip-over technique. (b) AFM image of the surface of the h-BN stack, after thermal annealing at T = 600 ˚C in Ar/H_2 gas flow for 10 hours. Root mean square (rms) obtained in the area indicated by the dashed square is 0.05 nm. (c) Line profile of the white dotted line in (b).

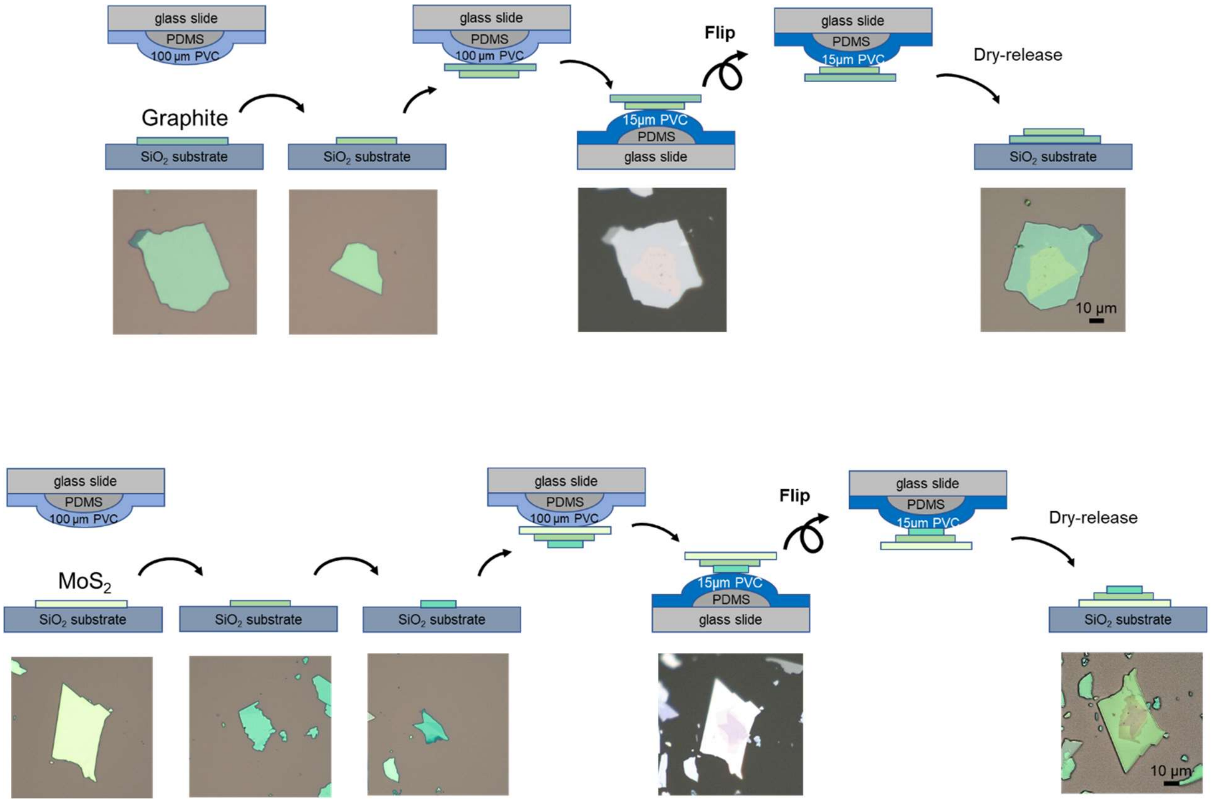

Fig. S5. Flip-over stacking of (a) graphite flakes and (b) MoS_2 flakes. Graphite and MoS_2 flakes were exfoliated onto SiO_2/Si substrates and picked up by a 100 µm PVC/PDMS stamp. The stacks were transferred to a 15 µm PVC/PDMS stamp at T = 95 ˚C. The flipped heterostructures were dry-released onto a SiO_2/Si substrate at T = 130 ˚C.

Comment on polymer-to-polymer transfer from thinner to thicker films

The transfer ratio was not zero even when transfer from thinner to thicker films was attempted, and the transfer ratio increased with temperature (~30% at T > 130 °C). It is possible that gravity or a temperature gradient across the PVC stamp was to some extent, responsible for this behavior. Expansion of the PDMS dome at high temperatures may also play a role.

Notes on the conditions required for successful polymer-to-polymer transfer

The optimal thickness and DOP content of the PVC films used in polymer-to-polymer transfer are now discussed, along with some other practical aspects of the technique.

(1) Film Thickness: The PVC film needs to be reasonably thick to prevent deformation upon being brought into contact with the PVC stamp. When the film was too thin (~1 μm), it was separated from the PDMS dome during stamp detachment. When this occurred, the transfer rate was drastically decreased. Therefore, we recommend using PVC films thicker than 10 μm.

(2) DOP content: We assumed that the optimal DOP content of the PVC film was ~20–40%, at which both strong adhesion and film flexibility was realized. When the DOP content was greater than 40%, the adhesion between the PVC and 2D flakes was somewhat reduced. Meanwhile, without the addition of DOP, the PVC lacked flexibility and tended to crack as the film thickness increased. Thus, although T_{pickup} and T_{release} can be modulated by tuning the plasticizer content, it is preferable to change T_{pickup} and T_{release} by varying the film thickness.

(3) Preservation of PVC stamp: The PVC stamp should be used just after being baked in a vacuum oven or stored in a vacuum desiccator. When the stamp was used after being left in the air for long periods of time, polymer-to-polymer transfer did not occur.

(4) Transfer temperature: Polymer-to-polymer transfer did not occur when the temperature was rapidly increased far above the optimal temperature. Higher temperatures did not always result in higher transfer ratios. In practice, the best approach is to start at a temperature slightly lower than the optimal temperature, and then gradually increase the stage temperature until the transfer occurs.

(5) Reuse of PVC stamp: The pickup force of the PVC stamp became a little weaker after repeated use of the PVC stamp. Thus, we recommend renewing PVC stamps on a regular basis to ensure reproducibility.
